# Supplementary material for: HIV prevalence and awareness among adults presenting for enrolment into a study of people at risk for HIV in Kisumu County, Western Kenya
Source: PLoS One. 2024 Jan 2;19(1):e0294860. doi: 10.1371/journal.pone.0294860 (PMC10760834; doi:10.1371/journal.pone.0294860)
Supplement: Attachment — Submitted filename: HIV prevalence and awareness among adults presenting for enrolment into a study of people at risk for HIV in Kisumu County - VO comments.doc [file pone.0294860.s001.doc]

HIV prevalence and awareness among adults presenting for enrolment into a study of people at risk for HIV in Kisumu County, Western Kenya

Review comments:

Lines 52 -54 should be pushed up to begin at line 46 for ease of flow in HIV statistical data.

Line 171 – according to table 1, newly diagnosed males stand at 41% and not 46% as indicated

Line 171 – the results on age are not consistent with the data on table 1. I know median data is reported but can the same info be added on the table?

Line 216: this is true but sample size for both surveys were different, it may be unfair to conclude that the findings are similar.

Table 1: under sex, was there any data collected from the LGBTQ population? The study was recruiting key pops and hence the sex results expected should be more than just male or female. Anal sex is reported but that can mean either MSM or females having anal sex with men?

Lines 222-224: this is already reported under conclusion. There are bits of repetition here that the authors can revise.

Line 231: on vertical transmission, it would be important to break down the newly-diagnosed data into age vs sex to better understand what age group would mostly be affected here. It may be all since the study age group was 18-35

Line 248: relates to comment on sex above – did the survey question for sexual orientation so that the LGBTQ population is covered? These are he key pops at highest risk
